# Does Stress within a Muscle Change in Response to an Acute Noxious Stimulus?

**DOI:** 10.1371/journal.pone.0091899

**Published:** 2014-03-13

**Authors:** Kylie Tucker, Paul W. Hodges, Wolbert Van den Hoorn, Antoine Nordez, François Hug

**Affiliations:** 1 School of Health and Rehabilitation Sciences, National Health and Medical Research Council (NHMRC) Centre of Clinical Research Excellence in Spinal Pain, Injury and Health, The University of Queensland, Brisbane, Queensland, Australia; 2 School of Biomedical Sciences, The University of Queensland, Brisbane, Queensland, Australia; 3 University of Nantes, Laboratory “Motricité, Interactions, Performance” (EA 4334), Nantes, France; Semmelweis University, Hungary

## Abstract

**Background:**

Altered muscle activation during pain is thought to redistribute stress within muscles and ultimately decrease the load on painful structures. However, change in muscle stress during pain has not been directly tested. The aim of the present study is to determine whether stress within muscle tissue is reduced during local acute experimental pain.

**Methods and Results:**

Ten participants attended 2 experimental sessions that each involved isometric knee extension tasks in 2 series of control trials and 1 series of test trials at ∼10%MVC. Shear elastic modulus was measured from vastus lateralis using a shear wave elastographic technique (Supersonic Shear Imaging). Prior to the test contractions, a bolus of hypertonic (Pain) or isotonic saline (No-pain) was injected into vastus lateralis. Pain intensity was 5.2±1.0 during the painful contractions. The intra-session repeatability of the shear elastic modulus determined between control trials was good (ICC: 0.95 and 0.99; SEM: 5.1 and 9.3 kPa for No-pain and Pain, respectively). Muscle shear elastic modulus did not change systematically during Pain or No-pain contractions (all main effects and interaction P>0.14). Examination of data for individual participants showed that stress either increased or decreased. If the absolute change in modulus is considered between the control and the test trials, the change during Pain (16.2±9.5 kPa) was double that observed with No pain (7.9±5.9 kPa; P = 0.046).

**Conclusion:**

This is the first study to directly determine the change in stress within a muscle (change in shear elastic modulus) during pain. We conclude that experimental pain induced by hypertonic saline does not induce a systematic reduction in muscle stress during a single-joint isometric task. Therefore, the changes in muscle activity reported previously during similar tasks are unlikely to systematically reduce load in the painful region. Whether the individual-specific increase and decrease are physiologically relevant or purposeful requires further investigation.

## Introduction

Motor strategy changes during pain in multi-joint tasks, as illustrated by limping with lower limb pain. Similarly, when pain is experimentally induced in one leg, force is redistributed to the non-painful leg during quiet stance [1] or bilateral plantar flexion [2]. These data imply a purposeful strategy to reduce load within the painful region. It is unclear if this adaptation occurs during an isometric single joint task where substantially fewer options are available for compensation.

Muscle activity is redistributed within and/or between synergist muscles during simple force matched tasks when pain is induced in [3]–[5] or near [6], [7] the contracting muscle(s). This redistribution of activity involves increased activity of some motor units (including the recruitment of new units that were not recruited to perform the same task before pain), and a coincident decreased activity of others (including the cessation of firing of some units) [5], [6], [8]. It has been proposed that this change in recruitment (potentially to preferentially activate motor units with a different force vector [9]–[12]) would redistribute stress within and/or between muscles and alters the load on painful structures with the outcome of reduced pain or protection of the painful part from further injury [7], [13], [14]. Although such changes in load in painful tissues during pain appear logical, this has not been directly tested.

Consistent with the hypothesis that the load distribution may be changed in pain, the redistribution of muscle activity during acute deep tissue pain is associated with changes in the direction of external force production [7]. However, neither electromyographic (EMG) nor external force recordings can provide conclusive evidence of altered load within the test muscle. Thus, it remains unclear whether the observed changes in motor unit recruitment [5], [6], [8] are sufficient to reduce stress in these acute pain studies. The alternate view is that the change in motor unit recruitment is unrelated to an attempt to change load and/or insufficient to change tissue load in single joint tasks.

Here we assessed whether stress within a muscle is altered during local acute pain, using an innovative shear wave elastographic technique. Supersonic Shear Imaging (SSI) quantifies the shear elastic modulus of a localised area of tissue by imaging the internal propagation of shear waves [15]. This technique provides reliable measurement of muscle shear elastic modulus during isometric contraction [16], with a strong linear relationship between shear elastic modulus and individual muscle force during non-fatiguing [17] and fatiguing contractions [18]. These results show that changes in muscle stress can be reliably estimated by changes in shear elastic modulus. Consequently, the use of SSI provides an opportunity to quantify if stress within muscle tissue is reduced during acute pain, thus probing for evidence of a mechanical outcome of the redistribution of motor unit activity within a muscle during acute pain.

The aim of the present study was to determine whether stress within muscle tissue is reduced during an acute experimental pain episode. We hypothesised that shear elastic modulus would reduce within the painful region, consistent with the proposition that pain adaptations aim to reduce pain and/or protect the painful part from further injury and/or pain [7], [13], [14].

## Methods

### Participants

Ten males (age: 28.2±5.8 years), all left leg dominant, participated in this study. All participants were healthy as examined by a physician, and provided written informed consent. The local ethics committee of Nantes Ouest IV approved the experiment (CPP-MIP-002; ref 21/12) and all procedures adhered to the Declaration of Helsinki. Each participant attended 2 experimental sessions (No-pain and Pain), separated by 5–7 days. One participant exhibited large muscle tremors during all contractions. As these tremors prevented an accurate measurement of the shear elastic modulus the data were excluded from analysis.

### Measurements

#### Knee extension torque

**Figure 1:**
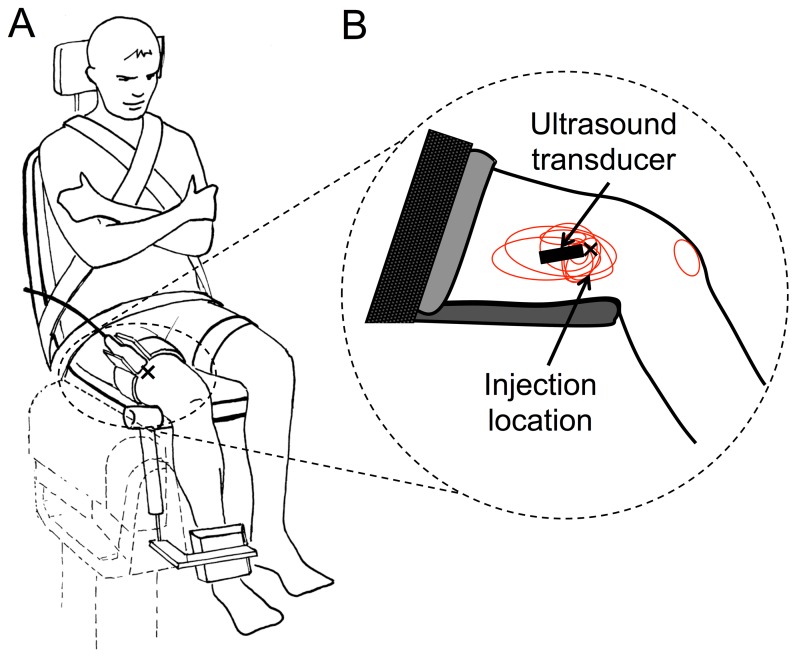
Experiential set up. A) The ultrasound transducer was placed on the distal part of the participant’s vastus lateralis muscle and held in position throughout the experiment by a custom-made support device. B) The location of saline injection (X) and region of reported pain for each individual (red) are shown relative to the ultrasound transducer placement on the upper leg.

Participants sat on an isokinetic dynamometer (Biodex System 3 Research, Biodex Medical, USA), with their torso reclined by 10° from upright and the right knee (test leg) flexed to 60° from the horizontal. Standard support straps were placed around the chest, pelvis and left (non-test) leg to minimise changes in body position throughout the experiment (Fig. 1). The axis of the dynamometer was aligned with the estimated axis of rotation of the right knee. Knee extension torque was digitized with a 16-bit analog-to-digital converter (Bagnoli 16, Delsys, USA) at 1000 Hz and low-pass filtered (6 Hz, 2^nd^ Butterworth filter).

#### Shear elastic modulus

An Aixplorer ultrasound scanner (version 4.2; Supersonic Imagine, Aix-en-Provence, France), coupled with a linear transducer array transducer (4–15 MHz, SuperLinear 15-4, Vermon, Tours, France) was used in SWE mode (musculo-skeletal preset) as in a manner similar to that described previously [15], [19]. Assuming a linear elastic behavior, the muscle shear elastic modulus (µ) was calculated as follow:
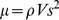
(1)


Where ρ is the muscle mass density (1000 kg.m^−3^) and Vs is the shear wave speed. As discussed previously [16], [20], the hypothesis of linear material is well accepted in studies of muscle elastography [15], [21], [22]. Maps of the shear elastic modulus were obtained at 1 Hz with a spatial resolution of 1×1 mm (Fig. 1B).

The ultrasound transducer was placed approximately 2/3 distally along the length of the vastus lateralis (VL), respecting the muscle fibre direction when possible. A brace attached to a custom-made device to support the ultrasound device was placed around the test leg. This device maintained a constant position of the ultrasound transducer throughout the experiment to avoid position changes with respect to the muscle over time. Care was taken to avoid inclusion of hyperechoic regions (areas of dense connective tissue) within the region of interest (ROI) of the ultrasound image as this may affect the accuracy of estimation of shear elastic modulus. The location of the ROI for calculation of the map within the test muscle (see Fig. 1B) was selected at the beginning of the experimental session, and remained consistent between conditions within a session. Force and SSI measurements were temporally synchronised with a voltage pulse related to the start of the SSI recording.

### Experimental Protocol

Prior to placement of the ultrasound transducer, participants performed two maximal isometric voluntary knee extension efforts for 3 s, separated by 90 s to allow recovery. The maximum torque was considered the best performance (maximum voluntary contraction; MVC). During the experimental task participants matched a knee extension target torque set at 10% of MVC with the exception of one participant who exhibited a high MVC torque. For this subject the feedback was set at 7% of MVC to prevent neuromuscular fatigue, which is known to develop more rapidly at higher absolute torques [23]. The relatively low force level was chosen for three reasons. First, this reduced the potential for neuromuscular fatigue that could interfere with interpretation of the data; second, this enabled comparison with changes in single motor unit discharge patterns that have been observed during pain with contractions at similar force levels [5], [7], [24]; third, greater changes in muscle activation and between-muscles compensations are more often observed at low contraction intensities (e.g. during pain, see systematic review [25]; during fatigue [26], [27]).

Participants performed 2 sessions on separate days. During each session, three series of ten 15-s contractions and two 60-s contractions at the target torque were performed, with 30-s rests between each contraction. The first two series of ten 15-s contractions (Control-1 and Control-2) were performed to determine the variation of the shear elastic modulus measure between contractions. During the second 60-s contraction (prior to the third series of ten 15-s contractions), an injection of either hypertonic saline (Pain) or isotonic saline (No-pain) was performed. The 60-s contraction was performed between the second control and the saline conditions, such that the injection could be performed with ultrasound guidance with the muscle in the contracted state. This ensured that the injected area was included in the region of the muscle from which the elastic modules was estimated in the subsequent test contractions. In addition, a 60-s contraction was also performed between Control-1 and Control-2, to ensure consistent conditioning of the muscle between conditions. A single MVC was performed immediately after the completion of the final contraction and was compared to the first MVCs to investigate evidence of muscle fatigue.

**Figure 2:**
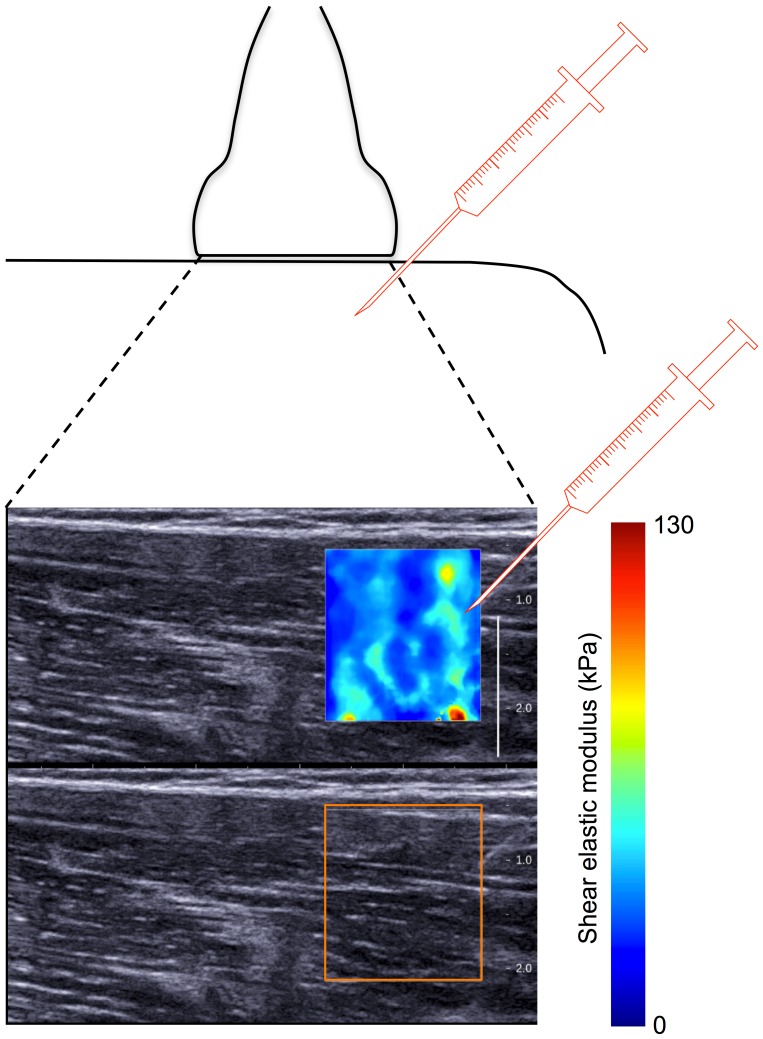
Injection of saline and example shear elastic modulus map. The approximate injection site and depth is shown relative to the ultrasound transducer. The map of shear elastic modulus (top) and B-mode ultrasound image (bottom) were collected simultaneously. A reflection of the needle is visible in the bottom ultrasound image.

The procedure was identical for both experimental sessions except that hypertonic saline (0.5 mL bolus 6.7% NaCl) was injected in one session to induce pain, and isotonic saline (0.7 mL bolus 0.9% NaCl) was injected as a control in the other. The isotonic saline was injected with larger volume to account for possible greater diffusion of water from surrounding tissue following the higher concentration hypertonic saline injection. Saline was injected approximately 15 s after commencement of the second 60-s contraction. In 5/10 participants, hypertonic saline was injected in the first session. Saline was injected (25–23 G×24–38 mm hypodermic needle) into the vastus lateralis at an angle ∼45° with ultrasound guidance (Fig. 2). The needle was inserted to a depth of ∼1.5 cm, which corresponds to the upper half of the analysed map. Participants rated pain intensity on an 11-point numerical rating scale (NRS), anchored with “no pain” at 0 and “maximum imaginable pain” at 10, at the mid point of each 15-s contraction. After completion of the experiment participants drew a surface representation of the region of pain, either directly onto their leg (and a photograph was taken) or, onto a photograph of their own leg [28]. As pain completely ceased (reported pain = 0/10) for all participants before completion of the ten 15-s contractions that followed the hypertonic saline injection, maps of shear elastic modulus were only considered for analysis from the first two 15-s contractions in this condition. For all participants pain was reported as greater than or equal to 2/10 during these contractions. For consistency, data from two 15-s contractions in the isotonic saline (No-pain) condition were considered for analysis. Three participants reported slight pain (1–2/10) during the first one to three 15-s contractions following injection of isotonic saline. As the isotonic saline condition was included to determine whether introduction of saline into the muscle tissue (as distinct from the effect of pain) alters shear elastic modulus measures, the two 15-s contractions that followed complete resolution of this pain were analysed for these participants.

### Additional Experiments

To confirm that the presence of saline alone does not alter resting muscle shear elastic modulus (and thus the y-intercept of the relationship between shear elastic modulus and stress) we performed an additional control study in 4 participants. SSI was used to quantify muscle shear elastic modulus at rest before and after injection of isotonic or hypertonic saline. Participants were seated as during the main protocol. Saline was injected with identical quantity into the distal portion of VL. SSI measurements were performed for 3×10 s rest periods before and immediately after the injection.

### Data Processing

Data were processed using Matlab (The Mathworks, Natick, MA, USA). SSI recordings were exported (Version 4.2, Supersonic Imagine, Aix en Provence, France) in “mp4” format and sequenced into images (portable network graphics lossless image compression). Image processing converted the colored map into shear elastic modulus values. Before analysis of the shear elastic modulus, the ROI was inspected for artefacts (e.g. areas of saturation of the shear elastic modulus measurement at 266 kPa, or no measurement (black) in the image). If artefacts were present in any of the images to be analysed within a session, the ROI was reduced in size to exclude the area of artefact from all images within that session. The mean±SD areas of the ROI used for the final analysis were 168±24, and 176±14 mm^2^ for Pain and No-pain sessions, respectively.

The middle 10 s (10 ultrasound images) of the 15-s contractions was used for analysis. Shear elastic modulus data and corresponding knee extension torque were averaged for each series of 15-s contractions separately (i.e. ten contractions for both of the control conditions and two contractions following the injection of either hypertonic or isotonic saline).

### Statistical Analysis

Statistics were performed in Stata (StataCorp LP, Texas, USA). To ensure normal distribution, data were transformed (log/square root) if Shapiro-Wilk test for normality was significant (*P*<0.05). First, the within-session repeatability of the shear elastic modulus measurement was calculated between Control-1 and Control-2 for the two sessions independently following recommendations of Hopkins [29]. As repeatability was very high (interclass correlations (ICC): pain session: 0.96; no-pain session: 0.99; standard error of measurement (SEM): Pain session: 9.9 kPa; No-pain session: 5.1 kPa), Control data were averaged over the 2 repetitions within each session for inclusion in the repeated measures ANOVA.

Then, torque and the mean shear elastic modulus were analysed with separate repeated measures analysis of variance (ANOVA), with Sessions (No-pain and Pain) and Conditions (Control, Saline) as within subject variables. Post hoc testing was conducted with Bonferroni correction. Torque was compared between MVC efforts using an ANOVA with Time (beginning and end) and Session (No pain and Pain) as within subject variables.

All data are reported as mean±SD unless stated otherwise. Significance level alpha was set at *P*<0.05.

## Results

### Knee Extension Torque

One participant was unable to complete the final MVC during the No-pain session because of muscle cramping, thus analysis of MVC is based on data from 8 participants. MVC (240±47 and 243±48 Nm for the Pain and No-pain sessions respectively) did not differ between sessions (main effect: Session P = 0.20) or between contractions performed before and after the experimental task (main effect: Time P = 1.00). The torque produced during the submaximal contractions was not different between session (main effect of Session: P = 0.85) and between conditions (main effect of Condition: P = 0.61).

### Pain

All participants reported pain intensity of greater than 2/10 at the beginning of contractions that followed hypertonic saline injection, and had recovered to 0/10 in all participants before completion of the ten series of contractions. During the first two 15-s contractions used for analysis the mean pain intensity was 5.2±1.0 (range: 3–6). The area of pain was confined to the region of the injection, except for one participant who reported an additional area of referred pain in the anterior knee region (Fig. 1B).

### Shear Elastic Modulus

When mean shear elastic modulus data were analysed for the group there was no systematic reduction during Pain (Control: 83.2±36.8 kPa, Pain: 94.7±35.3 kPa) or following the injection of non-painful isotonic saline (Control: 85.7±36.8 kPa, No-pain: 89.5±30.5 kPa) (main effect of Condition: P = 0.14, main effect of Session: P = 0.87, interaction Condition × Session: P = 0.22).

**Figure 3:**
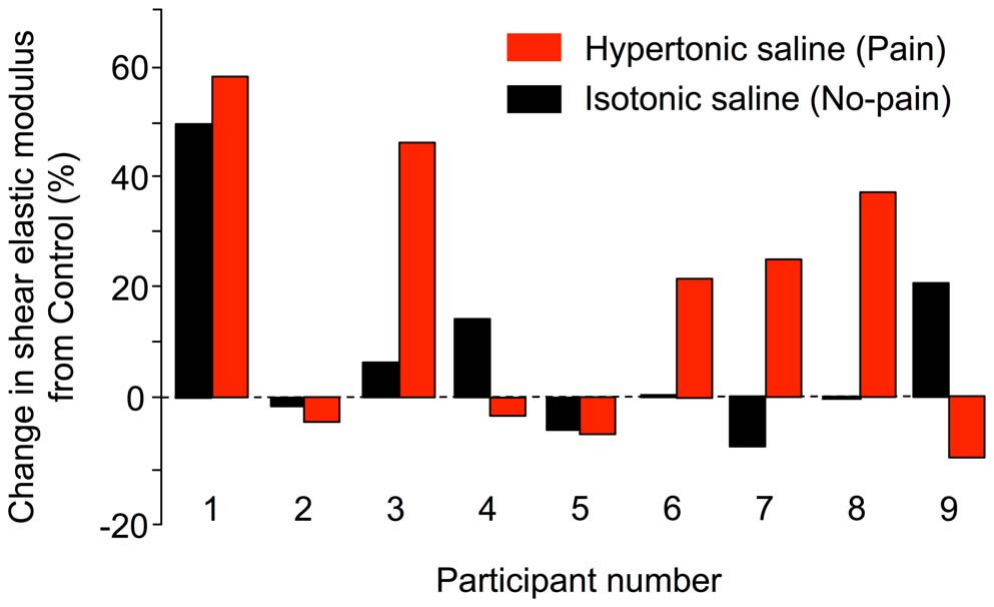
Change in shear elastic modulus from Control. Percentage of change in muscle shear elastic modulus from Control condition is depicted for both isotonic saline (No-pain: black) and hypertonic saline (Pain: red) condition. The absolute amplitude of change (not shown) was greater following the hypertonic saline injection, but whether shear elastic modulus increased or decreased differed between participants.

Examination of data for individual participants revealed individual variation in shear elastic modulus during pain. An increase of more than 10% was observed in 5/9 participants during pain, a decrease by more than 10% in 1/9 participant, and minor changes less than 10% were observed in the remaining 3 (Fig. 3). In view of this unexpected variation (with a propensity for increased rather than decreased stress) we undertook additional exploratory analyses of the data. If the absolute change in modulus is considered between the second control and the saline contractions, and a t-paired test performed, the change during Pain (16.2±9.5 kPa) was double that observed with No pain (7.9±5.9 kPa), P = 0.046.

### Additional Experiment

There was no change in the resting modulus in the 4 participants either following isotonic saline injection (−0.1±0.5 kPa), or following the hypertonic saline injection when pain was rated at 4.5±2.1/10 (−0.1±1.0 kPa). This confirms that the presence of saline alone does not alter resting muscle shear elastic modulus (and thus the y-intercept of the relationship between shear elastic modulus and stress).

## Discussion

Changes in muscle activity during an acute noxious stimulus (e.g. experimental pain) are hypothesised to alter the stress on painful structures [7], [13], [14], but this has been never been directly tested. Taking advantage of an innovative elastographic technique, the present study is the first to assess the effect of experimental pain on stress within a painful muscle. We provide evidence that stress within a painful muscle does not systematically decrease. Although unloading of a painful segment/muscle is observed in multijoint balance tasks [30], this same reduction of stress is not observed during acute pain in an isometric single joint task that present limited options for compensation. This is contrary to the predictions from earlier work [7], [13]. It is therefore important to consider that the variable change in stress may represent a more complex solution to protect the painful part.

Consistent with the hypothesis of decreased load on painful tissue, some studies report decreased gross myoelectric activity level within a painful muscle [31], [32]. However, this is not always observed, especially at low contraction intensity [33]–[35]. Increased muscle activity during similar painful tasks has also been reported [36], [37] and a shift in the relative activation of muscle regions has been observed using surface array electrodes during acute muscle pain [4] in a manner that was independent of the site of noxious stimulation to the muscle [3]. Interpretation of these results from surface EMG recordings is not straightforward, as the surface signal represents the summation of the activity of many motor units and the discharge behaviour of individual units has been shown to change in a variable manner during pain [38]. Discharge of single motor units has been shown to increase and decrease within a single fine-wire EMG recording zone and this varies between recording sites during acute experimental pain during single joint isometric tasks similar to that investigated in the current study [5]–[7]. Without direct measurement of muscle stress the outcome of the complex adaptations in muscle activation is difficult to predict.

Although muscle stress can be assumed to be less when the force output of the task is reduced [39] here we provide the first direct evidence that when the objective of the task is to maintain a constant force output in a single joint isometric task, there is no systematic reduction of load in the painful muscle. Our measurement of muscle stress provides unique insight into this debate as changes in EMG do not directly infer a change in muscle stress. This is because the relationship between EMG and stress is complicated by numerous physiological(e.g. fibre membrane properties, motor unit properties) and non-physiological (e.g. anatomical/geometrical properties of the muscle and EMG detection system properties, including crosstalk between muscles/regions of muscles, summation of action potentials from multiple motor units) factors [40], [41]. Whether the absence of systematic reduction of muscle stress in this task infers that the EMG changes are unrelated to a strategy to reduce stress, insufficient to unload the painful tissues, or represent a more complex solution to protect the painful part requires further consideration.

Although no systematic change in shear elastic modulus was observed, we did observe a change in this measure by >10% during pain in 6/9 participants. The non-systematic change in stress between individuals is consistent with non-systematic changes in the direction of external force production during acute deep tissue pain during a similar task [7]. The non-systematic change in muscle stress could be a consequence of the characteristics of the experimental task. This isometric, single-joint, low force task presents the nervous system with few degrees of freedom to modify yet maintain the goal of the task (matched knee extension torque). This may have restricted the potential for systematic redistribution of stress within and between-muscles, with a resultant small and variable (individual specific) modification of stress on the painful tissue. Multi-joint tasks have greater potential for compensation between segments. In that context the redistribution of muscle activity or stress may be systematic and predictable (e.g. redistribution of force between legs in bilateral lower limb tasks [1], [2]) or also demonstrate variation between individuals (e.g. increased motion at hip or knee to compensate for decreased spine motion secondary to experimental back pain [42]).

The method of pain induction with hypertonic saline might also complicate the interpretation of our findings. This is because participants do not systematically report that pain from hypertonic saline injection increases when the muscle is loaded with contraction. We have observed that approximately half of our participants report a small reduction (∼1–2/10) of pain during contraction, whereas others report no change or an increase. Thus, some participants may achieve “pain relief” by loading the tissue when pain is induced in this way, whereas others may not. If alternative methods for pain induction (e.g., injection of Nerve Growth Factor) can be shown to produce pain that consistently increases with muscle contraction this might show more consistent changes in muscle stress during pain.

The inclusion of a series of control contractions with injection of non-noxious isotonic saline enabled determination of the potential effect of saline/additional fluid in the muscle tissue on muscle stress. If the presence of saline (in the absence of pain) affected stress, this was expected to induce a consistent change in shear elastic modulus (i.e. either increase or decrease stress) in all participants. Rather, injection of isotonic saline induced no mean change in shear elastic modulus (Fig. 3). To further confirm that the presence of saline alone does not alter resting muscle shear elastic modulus (and thus the y-intercept of the relationship between shear elastic modulus and stress) we performed an additional control study in 4 participants where SSI was used to quantify muscle shear elastic modulus at rest before and after injection of isotonic and hypertonic saline. We found no change in resting modulus (−0.1±0.5 kPa following isotonic saline injection (no pain), and −0.1±1.0 for the hypertonic saline injection when pain was rated at 4.5±2.1/10.

### Conclusion

Investigation of changes in shear elastic modulus provided a first step towards evaluation of whether altered muscle activation is a purposeful adaptation by the nervous system to modify muscle stress. We provide evidence that during a simple, single joint task with the objective to maintain force, pain induced with hypertonic saline did not induce a systematic reduction of stress within the painful tissue. However, exploration of that data showed individual variation in behaviour that aligned with previous studies of external force direction during similar tasks. This individual variability requires further exploration to determine the possible physiological relevance. We conclude that the change in muscle activity during acute experimental pain in this single joint task is unrelated to, or insufficient to unload the painful tissues. Further work is required to determine if similar changes are observed in more complex tasks, when there is greater opportunity for compensation between muscles/segments such that the a painful region can be unloaded while maintaining the objective of the task.

## References

[1] Hirata RP, Arendt-Nielsen L, Graven-Nielsen T (2010) Experimental calf muscle pain attenuates the postural stability during quiet stance and perturbation. Clin Biomech 25: 931–937. 10.1016/j.clinbiomech.2010.06.001 20692746

[2] Hug F, Hodges P, Salomoni SE, Tucker KJ (2014) Insight into motor adaptation to pain from between-leg compensation. Eur J Appl Physiol In press. 10.1007/s00421-014-2840-y 24514948

[3] Falla D, Arendt-Nielsen L, Farina D (2009) The pain-induced change in relative activation of upper trapezius muscle regions is independent of the site of noxious stimulation. Clin Neurophysiol 120: 150–157. 19028440 10.1016/j.clinph.2008.10.148

[4] Madeleine P, Leclerc F, Arendt-Nielsen L, Ravier P, Farina D (2006) Experimental muscle pain changes the spatial distribution of upper trapezius muscle activity during sustained contraction. Clin Neurophysiol 117: 2436–2445. 16996301 10.1016/j.clinph.2006.06.753

[5] Tucker K, Butler J, Graven-Nielsen T, Riek S, Hodges P (2009) Motor unit recruitment strategies are altered during deep-tissue pain. J Neurosci 29: 10820–10826. 19726639 10.1523/JNEUROSCI.5211-08.2009 PMC6665526

[6] Tucker K, Larsson AK, Oknelid S, Hodges P (2012) Similar alteration of motor unit recruitment strategies during the anticipation and experience of pain. Pain 153: 636–643. 22209423 10.1016/j.pain.2011.11.024

[7] Tucker KJ, Hodges PW (2010) Changes in motor unit recruitment strategy during pain alters force direction. Eur J Pain 14: 932–938. 20378379 10.1016/j.ejpain.2010.03.006

[8] Minami I, Akhter R, Albersen I, Burger C, Whittle T, et al (2013) Masseter Motor Unit Recruitment is Altered in Experimental Jaw Muscle Pain. J Dent Res 92: 143–148. 23242229 10.1177/0022034512470832

[9] Riek S, Bawa P (1992) Recruitment of motor units in human forearm extensors. J Neurophysiol 68: 100–108. 1517816 10.1152/jn.1992.68.1.100

[10] ter Haar Romeny BM, Denier van der Gon JJ, Gielen CC (1982) Changes in recruitment order of motor units in the human biceps muscle. Exp Neurol 78: 360–368. 7140904 10.1016/0014-4886(82)90054-1

[11] Thomas JS, Schmidt EM, Hambrecht FT (1978) Facility of motor unit control during tasks defined directly in terms of unit behaviors. Exp Neurol 59: 384–397. 648610 10.1016/0014-4886(78)90230-3

[12] Yang D, Morris SF, Sigurdson L (1998) The sartorius muscle: anatomic considerations for reconstructive surgeons. Surg Radiol Anat 20: 307–310. 9894308 10.1007/BF01630610

[13] Hodges PW, Tucker K (2011) Moving differently in pain: a new theory to explain the adaptation to pain. Pain 152: S90–98. 21087823 10.1016/j.pain.2010.10.020

[14] Peck CC, Murray GM, Gerzina TM (2008) How does pain affect jaw muscle activity? The Integrated Pain Adaptation Model. Aust Dent J 53: 201–207. 18782363 10.1111/j.1834-7819.2008.00050.x

[15] Bercoff J, Tanter M, Fink M (2004) Supersonic shear imaging: a new technique for soft tissue elasticity mapping. IEEE Trans Ultrason Ferroelectr Freq Control 51: 396–409. 15139541 10.1109/tuffc.2004.1295425

[16] Nordez A, Hug F (2010) Muscle shear elastic modulus measured using supersonic shear imaging is highly related to muscle activity level. J Appl Physiol 108: 1389–1394. 20167669 10.1152/japplphysiol.01323.2009

[17] Bouillard K, Nordez A, Hug F (2011) Estimation of individual muscle force using elastography. PLoS One 6: e29261. 22229057 10.1371/journal.pone.0029261 PMC3244452

[18] Bouillard K, Hug F, Guevel A, Nordez A (2012) Shear elastic modulus can be used to estimate an index of individual muscle force during a submaximal isometric fatiguing contraction. J Appl Physiol 113: 1353–1361. 22984244 10.1152/japplphysiol.00858.2012

[19] Tanter M, Bercoff J, Athanasiou A, Deffieux T, Gennisson JL, et al (2008) Quantitative assessment of breast lesion viscoelasticity: initial clinical results using supersonic shear imaging. Ultrasound Med Biol 34: 1373–1386. 18395961 10.1016/j.ultrasmedbio.2008.02.002

[20] Lacourpaille L, Hug F, Bouillard K, Hogrel JY, Nordez A (2012) Supersonic shear imaging provides a reliable measurement of resting muscle shear elastic modulus. Physiol Meas 33: N19–28. 22370174 10.1088/0967-3334/33/3/N19

[21] Catheline S, Gennisson JL, Delon G, Fink M, Sinkus R, et al (2004) Measuring of viscoelastic properties of homogeneous soft solid using transient elastography: an inverse problem approach. J Acoust Soc Am 116: 3734–3741. 15658723 10.1121/1.1815075

[22] Dresner MA, Rose GH, Rossman PJ, Muthupillai R, Manduca A, et al (2001) Magnetic resonance elastography of skeletal muscle. J Magn Reson Imaging 13: 269–276. 11169834 10.1002/1522-2586(200102)13:2<269::aid-jmri1039>3.0.co;2-1

[23] Enoka RM, Duchateau J (2008) Muscle fatigue: what, why and how it influences muscle function. J Physiol 586: 11–23. 17702815 10.1113/jphysiol.2007.139477 PMC2375565

[24] Hug F, Hodges P, Tucker K (2013) Effect of pain location on spatial reorganisation of muscle activity. J Electromyogr Kinesiol in press. 10.1016/j.jelekin.2013.08.014 24055532

[25] Bank PJ, Peper CE, Marinus J, Beek PJ, van Hilten JJ (2013) Motor consequences of experimentally induced limb pain: a systematic review. Eur J Pain 17: 145–157. 22718534 10.1002/j.1532-2149.2012.00186.x

[26] Akima H, Foley JM, Prior BM, Dudley GA, Meyer RA (2002) Vastus lateralis fatigue alters recruitment of musculus quadriceps femoris in humans. J Appl Physiol (1985) 92: 679–684. 11796681 10.1152/japplphysiol.00267.2001

[27] Kouzaki M, Shinohara M (2006) The frequency of alternate muscle activity is associated with the attenuation in muscle fatigue. J Appl Physiol (1985) 101: 715–720. 16728513 10.1152/japplphysiol.01309.2005

[28] Tucker K, J, Fels M, Walker S, R, Hodges P, W (2013) Comparison of location, depth, quality and intensity of experimentally induced pain in six low back muscles. Clin J Pain in press. 10.1097/AJP.0000000000000026 25098553

[29] Hopkins WG (2000) Measures of reliability in sports medicine and science. Sports Med 30: 1–15. 10907753 10.2165/00007256-200030010-00001

[30] Hirata RP, Arendt-Nielsen L, Graven-Nielsen T (2010) Experimental calf muscle pain attenuates the postural stability during quiet stance and perturbation. Clin Biomech (Bristol, Avon) 25: 931–937. 10.1016/j.clinbiomech.2010.06.001 20692746

[31] Graven-Nielsen T, Svensson P, Arendt-Nielsen L (1997) Effects of experimental muscle pain on muscle activity and co-ordination during static and dynamic motor function. Electroencephalogr Clin Neurophysiol 105: 156–164. 9152211 10.1016/s0924-980x(96)96554-6

[32] Ciubotariu A, Arendt-Nielsen L, Graven-Nielsen T (2004) The influence of muscle pain and fatigue on the activity of synergistic muscles of the leg. Eur J Appl Physiol 91: 604–614. 14685868 10.1007/s00421-003-1026-9

[33] Hodges PW, Ervilha UF, Graven-Nielsen T (2008) Changes in motor unit firing rate in synergist muscles cannot explain the maintenance of force during constant force painful contractions. J Pain 9: 1169–1174. 18755636 10.1016/j.jpain.2008.06.012

[34] Madeleine P, Arendt-Nielsen L (2005) Experimental muscle pain increases mechanomyographic signal activity during sub-maximal isometric contractions. J Electromyogr Kinesiol 15: 27–36. 15642651 10.1016/j.jelekin.2004.06.006

[35] Farina D, Arendt-Nielsen L, Merletti R, Graven-Nielsen T (2004) Effect of experimental muscle pain on motor unit firing rate and conduction velocity. J Neurophysiol 91: 1250–1259. 14614105 10.1152/jn.00620.2003

[36] Del Santo F, Gelli F, Spidalieri R, Rossi A (2007) Corticospinal drive during painful voluntary contractions at constant force output. Brain Research 1128: 91–98. 17134682 10.1016/j.brainres.2006.09.039

[37] Fadiga L, Craighero L, Dri G, Facchin P, Destro MF, et al (2004) Corticospinal excitability during painful self-stimulation in humans: a transcranial magnetic stimulation study. Neurosci Letters 361: 250–253. 10.1016/j.neulet.2003.12.016 15135940

[38] Tucker KJ, Hodges PW (2009) Motoneurone recruitment is altered with pain induced in non-muscular tissue. Pain 141: 151–155. 19095357 10.1016/j.pain.2008.10.029

[39] Svensson P, Arendt-Nielsen L, Houe L (1996) Sensory-motor interactions of human experimental unilateral jaw muscle pain: a quantitative analysis. Pain 64: 241–249. 8740600 10.1016/0304-3959(95)00133-6

[40] Farina D, Merletti R, Enoka RM (2004) The extraction of neural strategies from the surface EMG. J Appl Physiol 96: 1486–1495. 15016793 10.1152/japplphysiol.01070.2003

[41] Hug F (2011) Can muscle coordination be precisely studied by surface electromyography? J Electromyogr Kinesiol 21: 1–12. 20869882 10.1016/j.jelekin.2010.08.009

[42] Smith M, Coppieters MW, Hodges PW (2005) Effect of experimentally induced low back pain on postural sway with breathing. Exp Brain Res 166: 109–117. 16032406 10.1007/s00221-005-2352-4

